# The Hepatitis B Virus X Protein Inhibits Thymine DNA Glycosylase Initiated Base Excision Repair

**DOI:** 10.1371/journal.pone.0048940

**Published:** 2012-11-08

**Authors:** Maarten A. A. van de Klundert, Formijn J. van Hemert, Hans L. Zaaijer, Neeltje A. Kootstra

**Affiliations:** 1 Department of Blood-borne Infections, Center for Infection and Immunity Amsterdam (CINIMA), Sanquin, Amsterdam, The Netherlands; 2 Department of Experimental Immunology, CINIMA, Academic Medical Center, University of Amsterdam, Amsterdam, The Netherlands; 3 Laboratory of Experimental Virology, Department of Medical Microbiology, CINIMA, Academic Medical Center, University of Amsterdam, Amsterdam, The Netherlands; 4 Laboratory of Clinical Virology, Department of Medical Microbiology, Academic Medical Center, University of Amsterdam, Amsterdam, The Netherlands; Yonsei University College of Medicine, Republic Of Korea

## Abstract

The hepatitis B virus (HBV) genome encodes the X protein (HBx), a ubiquitous transactivator that is required for HBV replication. Expression of the HBx protein has been associated with the development of HBV infection-related hepatocellular carcinoma (HCC). Previously, we generated a 3D structure of HBx by combined homology and ab initio *in silico* modelling. This structure showed a striking similarity to the human thymine DNA glycosylase (TDG), a key enzyme in the base excision repair (BER) pathway. To further explore this finding, we investigated whether both proteins interfere with or complement each other’s functions. Here we show that TDG does not affect HBV replication, but that HBx strongly inhibits TDG-initiated base excision repair (BER), a major DNA repair pathway. Inhibition of the BER pathway may contribute substantially to the oncogenic effect of HBV infection.

## Introduction

Infection with hepatitis B virus (HBV) is predominantly cleared in adults, but particularly in younger patients it often leads to chronic infection. As a result of the ongoing viral replication and the immunological response to the infection, about 25% of chronically infected patients develop liver cirrhosis, inflammation and, ultimately, hepatocellular carcinoma (HCC). Although an effective vaccine is available, an estimated 350 million people are currently chronically infected with HBV, resulting in an estimated 600.000 deaths per year.

The HBV genome encodes a ubiquitous transactivator termed the HBV X protein (HBx), which is essential for HBV replication *in vivo*. Various lines of evidence indicate that HBx is at least partially responsible for the oncogenic effects of chronic HBV infection [1], [2]. For instance, HBx is often expressed from integrated parts of the HBV genome in HCC tissue [1], [3], and mice expressing HBx in their liver either develop HCC spontaneously [4] or display increased susceptibility to hepatocarcinogens [5]. Also, HBx was shown to impede the nucleotide excision repair (NER) pathway [6]–[8].

Although the exact function of the HBx protein is still unclear, several functions can be distinguished. HBx acts as a transactivator, which increases the transcription of mRNAs from the covalently closed circular DNA (cccDNA) of HBV by altering its epigenetic context [9]. Transactivational effects are guided by associations between HBx and various transcription factors [10]–[12]. HBx associates with DDB1 [13]–[15], and although this interaction is dispensable for the transactivation by HBx [16], it is essential for HBV replication *in vitro* [17] and for establishing HBV infection in animal models [18], [19].

Previously we reported a striking similarity between the predicted structure of the HBx protein and the central domain of DNA glycosylases [20]. DNA glycosylases initiate base excision repair (BER) by glycosylation of the bond between a mismatched base and the phosphate backbone of a DNA strand. Among the proteins showing prominent 3D similarity to HBx is the human thymine DNA glycosylase (TDG). Besides its function in BER, TDG is a key regulator of transcription and involved in the epigenetic regulation of DNA in the context of both transcription and DNA repair [21].

In this paper, we establish a functional relation between HBx and TDG, substantiating the structural homology between HBx and the central domain of a member of the DNA glycosylase family. Our results indicate that TDG does not affect HBV replication *in vitro*, but we found that HBx strongly inhibits TDG-initiated BER.

## Materials and Methods

### Cell Culture and Transfection

HEK 293 cells were maintained in Dulbecco’s Modified Eagle Medium without HEPES (DMEM)(LONZA, Basel, Swizerland) supplemented with 10% heat inactivated fetal calf serum, penicillin (100 U/mL) and streptomycin (100 µg/mL) (Gibco Pen Strep). HepG2 cells were maintained in William’s Medium E w/o L-Gln (LONZA, Basel, Swizerland), supplemented with 10% v/v inactivated fetal calf serum, 2 mM L-glutamin (LONZA, Basel, Swizerland), penicillin (100 U/mL), streptomycin (100 µg/mL) and 5 uM Dexamethasone (Sigma Aldrich). HepG2 cells were maintained in a humidified 10% CO_2_ incubator at 37°C and HEK 293 cells were maintained in a humidified 5% CO_2_ incubator at 37°C. Twenty-four hours before transfection the cells were plated into 6- or 96-well culture plates. The calcium phosphate method was used for transfection. Briefly, plasmid DNA was diluted in 42 mM HEPES pH 7.2 and 2.5 M CaCl_2_ was added to a final concentration of 0.30 M CaCl_2_. The DNA mixture was added to an equal volume of 2× HEPES buffered saline (HBS)(275 mM NaCl, 10 mM KCl, 1.4 mM Na_2_HPO_4_, 42 mM HEPES pH 7.2) and after a 15 minute incubation at room temperature the mixture was added to the cells. Cells were cultured over night in a humidified 3% CO_2_ incubator at 37°C and subsequently the medium was replaced. For the transfection of HepG2 cells, a construct expressing GFP was routinely transfected in parallel to asses that the transfection efficiency was >5%. Cell cultures were maintained in a humidified incubator at 37°C supplemented with 5% (HEK 293) or 10% (HepG2) CO_2_.

### Expression Vectors

The R9 vector, containing a 1.2× overlength HBV DNA genome (subtype *adw*) in a pGEM 7zf+ backbone, was kindly provided by Dr. Baumert [22], [23]. To create an R9 vector lacking HBx expression (R9ΔX), the Glu87→STOP and Met103→Arg mutations were introduced by site directed mutagenesis [22]. To generate mutations in both the complete and partial HBx ORF, the partially redundant part of the vector was removed by *Pst1* digestion and ligated in the multiple cloning site of pcDNA 3.1A (−). The pGEM 7zf+ backbone vector containing the large part of the R9 HBV genome was re-ligated. The required mutations in both vectors were introduced sequentially by targeted mutagenesis (QuikChange II XL Site-Directed Mutagenesis Kit, Bio connect (Agilent Technologies)), according to the manufacturer’s instructions. Primer pairs used to generate a stop codon (Glu87→Stop) were 5′ ACCACCGTGAACGCCCATTAGATCCTGCCCAAGGTCTTA 3′ and 5′ TAAGACCTTGGGCAGGATCTAATGGGCGTTCACGGTGGT 3′ and the primers used to remove an alternative start codon (Met103→Arg) were 5′ GGACTCTTGGACTCCCAGCAAGGTCAACGACCGACCTTGAGG 3′ and 5′ CCTCAAGGTCGGTCGTTGACCTTGCTGGGAGTCCAAGAGTCC 3′ (substituted nucleotides in the primers are underlined). The presence of the substitutions was detected using AlwN1- and BsrD1 restriction and verified by sequencing. Subsequently, the small R9ΔX fragment was removed from the pcDNA 3.1A (−) vector by *Pst1* digestion and ligated into the *Pst1* site of the partial R9ΔX vector.

The HSV-tagged HBx expression vector pHSV-HBx was generated by PCR amplification (Expand High Fidelity TAQ, Roche) of the HBx gene from the R9 vector using a forward primer containing the HSV-tag: 5′ GCAGAATTCATGAGCCAGCCAGAACTCGCTCCTGAAGACCCAGAGGATGCTGCTAGGCTGTGCTGCC 3′ and reverse primer: 5′ TCCGGTACCTTAGGCAGAGGTGAAAAAGTTGC 3′. The obtained PCR product was digested with EcoR1 and Kpn1 and ligated in pcDNA 3.1A (−) multiple cloning site.

For the cloning of TDG, total RNA was isolated from HEK 293T cells using the RNeasy mini kit (Qiagen, Hilden, Germany) and cDNA was prepared using SuperScript™ First-Strand Synthesis System for RT-PCR (Invitrogen). For the construction of the Myc-tagged TDG expression vector (pMyc-TDG), TDG was cloned from HEK 293T cDNA by PCR using the Expand High Fidelity PCR and the following primer pair: forward primer containing the Myc-tag 5′ GCAGAATTCATGGAGCAGAAACTCATCTCTGAAGAGGATCTGGAAGCGGAGAACGCGGGC 3′ and reverse primer 5′ CTCGGATCCTCAAGCATGGCTTTCTTCTTCC 3′. The obtained PCR product was digested with EcoR1 and BamH1 respectively, and ligated in the multiple cloning site of pcDNA 3.1A (−).

### HBV Replication Assay

HepG2 cells were seeded in 6 well plates to reach a confluence of 30–40% at the moment of transfection. The HepG2 cells were transfected with 1 ug of the R9 or R9ΔX construct per well using calcium phosphate transfection as described above. Increasing concentrations of pHSV-HBX and pMyc-TDG were cotransfected as indicated. After over night incubation, the medium was replaced and cells were maintained in a humidified incubator at 37°C supplemented with 10% CO_2_ for 7 days. HepG2 cells were washed with PBS and harvested by trypsin digestion at 37°C for 7 minutes. Trypsin (LONZA, Basel, Swizerland) was inactivated by addition of fresh culture medium and cells were washed with PBS (LONZA, Basel, Swizerland). Cell pellets were lysed in 1 ml iso-osmotic lysis buffer (140 mmol/L NaCl, 1.5 mmol/L MgCl2, 50 mmol/L Tris-HCl [pH 8.0]) containing 0.5% Nonidet P-40) for 30 minutes on ice. To quantify capsid-associated HBV DNA, cell nuclei were pelleted at 400 g. Supernatants were harvested and remaining cell debris was removed by 10 minute centrifugation at 21,000 g. Remaining non-encapsidated viral DNA was removed from 200 µl cleared lysate by 1 × nuclease treatment for 45 minutes (Nuclease Mix, GE Healthcare Biosciences). Subsequently encapsulated viral DNA was purified using the Nucleospin Blood Kit (BIOKE) according to the manufacturer’s instructions. HBV DNA copy number was quantified by qPCR detecting a part of the core gene using forward primer 5′ GACCACCAAATGCCCCTAT 3′ and the reverse primer 5′ CGAGATTGAGATCTTCTGCGAC 3′ and the SYBR Green I Master (Roche) using the LightCycler® 480 system (Roche). The following program was used for qPCR: 10 min 95°C, followed by 50 cycles of 10 sec 95°C, 20 sec 59°C, 30 sec 72°C with a single acquisition during the 72°C step. For quantification of the HBV DNA copy number, the HBV core PCR fragment was cloned in the pGEM®-T Easy vector (Promega) and serial dilutions of this vector were used as a standard curve in each run. Quantification was performed using Roche’s LightCycler® relative quantification software (release 1.5.0).

**Figure 1:**
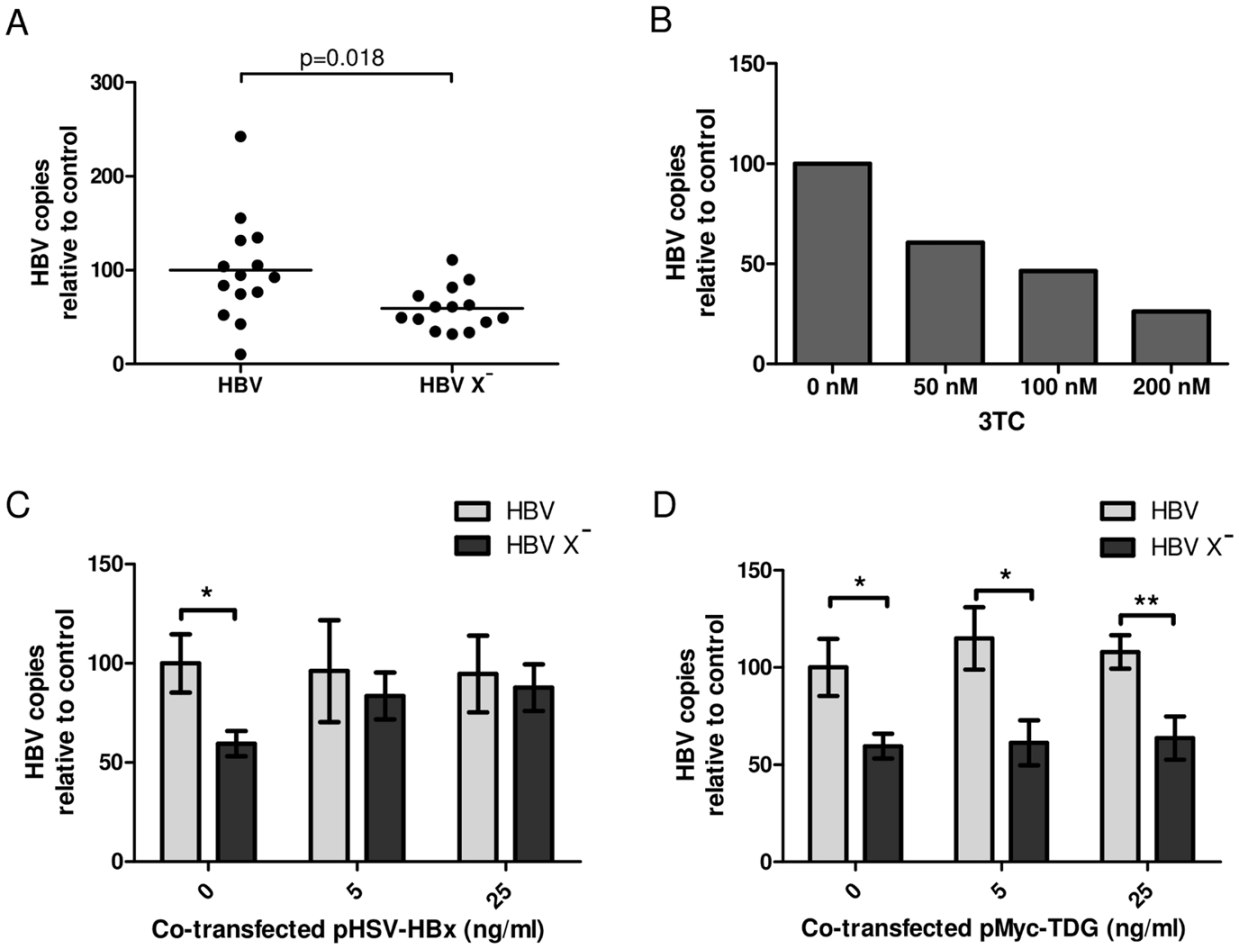
Replication of HBV X^-^ is rescued by co-expression of HBx but not TDG. (**A**) HepG2 cells were transfected with the R9 or R9ΔX vector to induce HBV replication in the presence (HBV) or absence (HBV X^−^) of HBx. (**B**) Addition of lamivudine to the cultures inhibited production of capsid-associated HBV DNA, indicating selective amplification of progeny DNA (**C**) Production of capsid-associated HBV DNA by R9ΔX was restored to wild-type levels by co-transfection of pHSV-HBx. (**D**) Co-expression of Myc-TDG does not affect HBV replication regardless of the presence of HBx. The average and standard deviation of the HBV DNA copy number of seven independent experiments is given. *p<0.05, **p<0.01. Significance was determined with a two-sided student’s T test.

### BER Assay

The BER assay was adapted from [24]. The pGL3-control vector (Promega, Madison, USA), containing a SV40 promoter driven luciferase reporter, was modified by insertion of an annealed primer pair with two cohesive *Nco*1 ends containing a *EcoR*1 and *Pst*1 sites downstream of the luciferase start codon, into the *Nco*1 site of pGL3. For the BER assay, the pG/C (positive control), pG/T (mismatch) and pA/T (negative control) vectors were generated by ligation of annealed primer pairs using 1.55 pmol *Eco*R1 and *Pst*1 digested vector, 15.5 pmol annealed primer pair and 400 units T4 DNA ligase (New England Biolabs) in a final volume of 500 µl. The pA/T, pG/C and pG/T vectors respectively contained one nucleotide difference resulting in a stop codon at amino acid position 13 for pA/T, which served as a negative control in the assay; a tryptophan at amino acid position 13, which served as a positive control in the assay; a G/T mismatch in pG/T, which encodes a stop codon or a tryptophan, depending on whether the vector was repaired or not. The ligation product was purified using the GFX PCR DNA and gel band purification kit (GE Healthcare) and used for transfection. Transfection of HEK 293 cells was performed in a 96 well plate using 50 ng pG/T, pA/T or pG/C combined with the indicated amounts of pHSV-HBx or pMyc-TDG in 4-fold.

**Figure 2:**
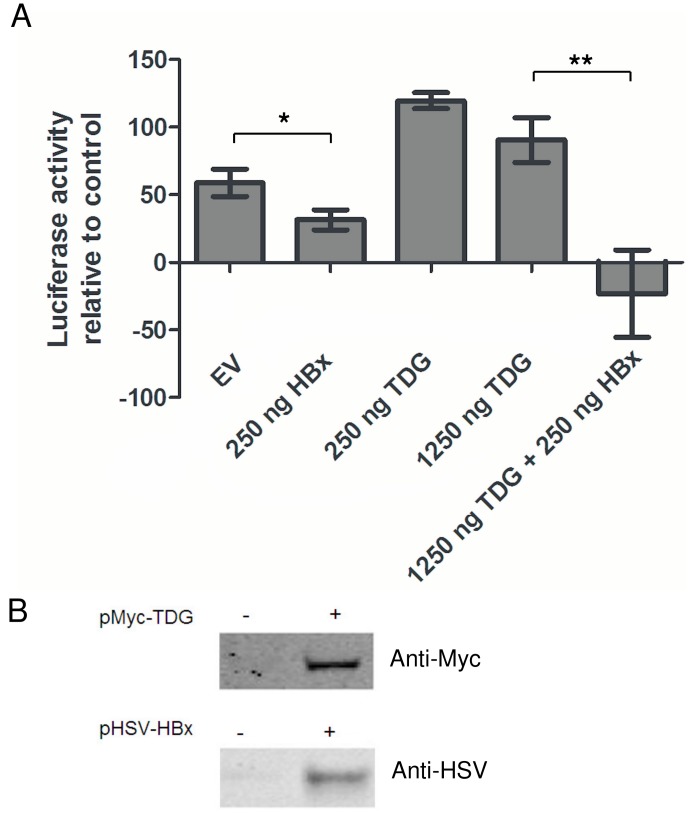
Base excision repair is inhibited by HBx. The effect of HBx and TDG on BER activity is given as the ratio between the luciferase activity of pG/T (mismatch) and pG/C (positive control) vectors after subtraction of the activity of the pA/T (negative control) vector. The activity of the pG/T vector was significantly less than the activity of the positive control, while co-transfection of pMyc-TDG restored the activity of the pG/T mismatch vector. Co-transfection of pHSV-HBx resulted in a reduction of BER activity, regardless of cotransfection of pMyc-TDG. Each bar depicts the mean and SEM of four measurements. DNA concentrations are expressed as ng/ml. EV: empty control vector. (B) Myc-TDG and HSV-HBx expression in HEK 293T cells was confirmed by Western blotting. *p<0.05, **p<0.01.

Twenty-four hours after transfection cells were lysed by addition of 25 ul luciferase substrate in concentrated lysis buffer containing 0.83 mM ATP, 0.83 mM luciferine-D, 18.7 mM MgCl_2,_ 0.78 µM Na_2_H_2_P_2_O_7_, 38.9 mM Tris pH 7.8, 0.39% glycerol, 0.03% triton X-100 and 2.6 µM dithiothreitol. Luciferase activity was assessed using a luminometer (Berthold, Bad Wildbad, Germany). The activity of the pA/T (negative control) vector was subtracted from the activity of the pG/C and pG/T vectors, and the activity of the pG/T vector relative to the pG/C vector was taken as the measure for BER activity.

### Western Blotting

HEK 293T cells were cultured in 6 well plates and transfected with 1 ug of pMyc-TDG or pHSV-HBx per well. After trypsin digestion, the cells were washed with PBS and centrifuged at 400 g for 10 minutes. The cell pellet was lysed in 1 ml of RIPA lysis buffer (150 mM NaCl, 1% Triton X-100, 0.5% sodium deoxycholate, 0.1% SDS, 50 mM Tris, pH 8.0) supplemented with Complete® EDTA free protease inhibitor (Roche). The lysate was denatured at 70°C for 10 minutes in 1× NuPAGE LDS sample buffer (Invitrogen) and 0.1 M DTT. Proteins were separated by electrophoresis on a 10% Bis-Tris gel (NuPAGE 10% Bis–Tris precast gel) together with the Odyssey Protein Weight Marker (LI-COR, Lincoln, NE, USA) using MES SDS running buffer (Invitrogen). Subsequently, proteins were transferred to a nitrocellulose membrane (Protran, Schleicher & Schuell, Dassel, Germany; 2 hours 150V) using NuPAGE transfer buffer. Blots were stained over night at 4°C in PBS (Gibco) supplemented with 0.01% Tween 20 (Merck) and 1% Protifar (Nutricia, Schiphol, The Netherlands) with a monoclonal mouse anti-c-Myc antibody (1∶5.000; Calbiochem, San Diego, CA, USA) or mouse anti-HSV antibody (1∶1000; Novagen). IRDye 800CW conjugated goat anti-mouse IgG (1∶15,000; 926–32210, LI-COR, Lincoln, NE, USA) was used as secondary antibody to visualize the proteins using the Odyssey infrared image system (LI-COR).

### Statistical Analysis

Statistical analysis was performed using GraphPad Prism (version 5). Significance of differences in HBV copy numbers and the effects of TDG and HBx on BER efficiency were determined by Student T test.

## Results

### TDG does not Affect HBV Replication

First we established whether we could measure an effect of HBx on HBV replication in HepG2 cells. The R9 construct harbouring a 1.2× overlength HBV genome and a mutant R9 construct (R9Δx) not expressing the HBx protein, were transfected in HepG2 cells. In line with other research [25], HBV not expressing HBx was hindered in its replication in HepG2 cells and produced 41% less HBV DNA (Fig. 1A). To ensure that the DNase treatment of the samples efficiently removed plasmid DNA containing the 1.2× overlength HBV genome, the HBV polymerase inhibitor lamivudine (3TC) was added to parallel cultures as a control. HBV DNA production was inhibited by lamivudine in a dose-dependent manner, indicating selective amplification of progeny DNA (Fig. 1B).

To assess the effect of TDG on HBV replication, the R9 and R9Δx vectors were co-transfected with increasing concentrations of plasmids expressing either HSV-tagged HBx (pHSV-HBx) or Myc-tagged TDG (pMyc-TDG). Indeed, replication of R9Δx could be rescued to wild-type level by co-transfection of pHSV-HBx (Fig. 1C). Co-transfection of pMyc-TDG could not rescue replication of the mutant and did not affect replication of the wild-type virus expressing HBx from its genome (Fig. 1D).

### HBx Inhibits (TDG initiated) Base Excision Repair

As TDG is a key enzyme in the Base Excision Repair (BER) pathway, we investigated whether HBx could influence TDG initiated repair of a G/T mismatch. We used the glycosylase assay established by Li et al. [24] with some minor adjustments (see materials and methods).

The pG/C (positive control), pG/T (mismatch) and pA/T (negative control) vectors were transfected into HEK 293 cells and luciferase activity was assessed 24 hours after transfection. The pA/T vector showed only low level of luciferase activity as compared to the positive control pG/C vector. We observed an increased luciferase activity of the mismatched pG/T vector as compared to the negative control, indicating that the G/T mismatch is repaired in HEK293 cells to some extent. To analyse the effect of HBx on TDG initiated repair of a G/T mismatch, pHSV-HBx or pMyc-TDG were co-transfected with the pG/C, pA/T and pG/T vectors in HEK293 cells and luciferase activity was analysed 24 hours after transfection. In figure 2A the effect of HBx and TDG on BER activity is given as the ratio between the luciferase activity of pG/T and pG/C vectors corrected for the background luciferase activity of pA/T under the same condition.

Activity of the mismatched vector could be restored to the same level as the positive control by co-transfection of pMyc-TDG (Fig. 2A), proving BER is essential for the activity of the pG/T vector and confirming the rate-limiting role of TDG in BER [26], [27]. When pHSV-HBx was co-transfected with the mismatched vector, the luciferase activity was inhibited (Fig. 2; p = 0.036) indicating that HBx inhibits BER of the mismatched pG/T vector in HEK 293 cells. When both pHSV-HBx and pMyc-TDG were cotransfected with the mismatched vector luciferase activity was also inhibited (Fig. 2A), indicating that HBx interferes with TDG initiated BER.

As both HBx [28] and TDG [29] can form homodimeres, we investigated whether HBx inhibits TDG initiated BER through direct interaction between the two proteins by means of co-immunoprecipitation and fluorescence microscopy of GFP fusion constructs. Our results indicate there is no direct interaction between the two proteins (data not shown). Using western blotting we established that TDG is not degraded in the presence of HBx (data not shown). Efficient expression of Myc-TDG and HSV-HBx in HEK 293T cells was confirmed by Western blotting (Fig. 2B).

## Discussion

Previously we generated a model of the tertiary structure of the HBV X protein by *in silico* modelling using I-Tasser [20]. The model was validated by successful *in silico* docking to known HBx binding partners, but no crystal structure of HBx is available to confirm our model. Querying the PDB database for proteins with similar structure, we found that our model of HBx showed striking similarity with the central domain of various members of the MUG family of DNA glycosylases, which are the key enzymes of the base excision repair pathway. Scoring high among the different glycosylases was the human thymine DNA glycosylase (TDG).

In this study we investigated a functional relation between HBx and TDG. Our results confirm the replication-enhancing role of HBx *in vitro* when replication in HepG2 cells was initiated by transfection of a 1.2×overlength HBV clone that was either capable of or deficient in HBx production. In the same model, we show that HBx, but not TDG could rescue replication of the virus deficient in HBx expression. TDG did not affect the replication of wildtype HBV either, indicating that TDG does not play a direct role in HBV replication. We observed that HBx strongly inhibits the TDG initiated base excision repair of a G/T mismatch in a manner not involving direct interaction between HBx and TDG. Inhibition of BER by HBx could be beneficial to HBV resulting in mutations in the cccDNA pool to allow selection of escape mutants, for instance when infected hepatocytes are subjected to adaptive immune responses. Indeed, an increase in the HBV mutation rate is seen when HBV viraemia is suppressed in the course of chronic infection [30], [31].

Inhibition of BER may contribute to the chemotherapy resistant phenotype of HBx positive HCC [32], considering that the cytotoxicity of 5-fluorouracil (5-FU) is mediated by TDG-initiated BER [33], and that HBV replication [34] as well as HBx expression [35], [36] have been demonstrated to induce resistance to 5-FU. Hence, it should be considered that treatment of HBx positive HCC with a DNA damaging chemotherapeutic may be more effective when the agent induces damage that is cytotoxic in the absence of BER.

Because there is no direct interaction between HBx and TDG, it seems likely that HBx interferes with BER by either degrading or interacting in a dominant negative fashion with one of the proteins further downstream the BER pathway. Deregulation of DNA repair may benefit the viral replication. Indeed, the use of DNA-damaging agents which disrupt/activate DNA-repair pathways, is strongly associated with reactivation of HBV infections. Although such reactivations are also observed when the immune system is suppressed [37], they are more common [38] and occur earlier [39] when therapy also induces DNA damage. Moreover, the DNA damaging agent doxorubicin strongly induces HBV replication in vitro in the absence of an immune response [39].

In conclusion, our finding that the HBx protein inhibits BER substantiates our hypothesis that there is a structural homology between HBx and the central domain of TDG. The inhibition of BER by HBx might contribute to the oncogenic effect of chronic HBV infection.
